# Supplementary material for: Vitamin D Modified DSS-Induced Colitis in Mice via STING Signaling Pathway
Source: Biology (Basel). 2025 Jun 18;14(6):715. doi: 10.3390/biology14060715 (PMC12190092; doi:10.3390/biology14060715)
Supplement: Supplementary file 1 [file biology-14-00715-s001.zip › Supplementary Materials S1: 16s-report of gut microbiota/Groups/diff_analysis/RF/Family_Groups.RFimportance.pdf]

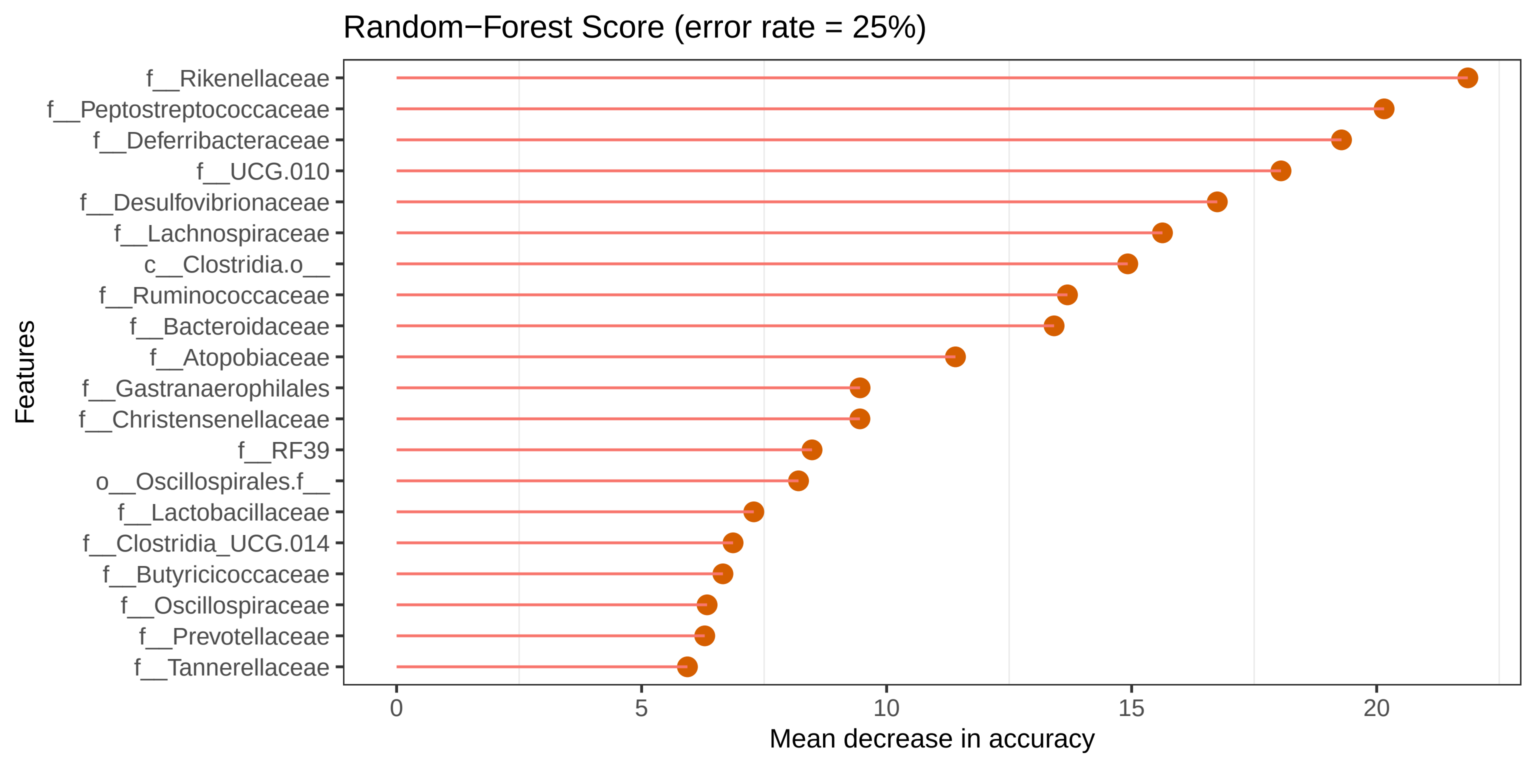
Random−Forest Score (error rate = 25%)
f__Rikenellaceae
f__Peptostreptococcaceae
f__Deferribacteraceae
f__UCG.010
f__Desulfovibrionaceae
f__Lachnospiraceae
c__Clostridia.o__
f__Ruminococcaceae
f__Bacteroidaceae
f__Atopobiaceae
f__Gastranaerophilales
f__Christensenellaceae
f__RF39
o__Oscillospirales.f__
f__Lactobacillaceae
f__Clostridia_UCG.014
f__Butyricicoccaceae
f__Oscillospiraceae
f__Prevotellaceae
f__Tannerellaceae
Features
0
5
10
15
20
Mean decrease in accuracy
